# An endolysosome pathway specific polygenic risk score for Alzheimer Disease predicts cell‐type specific transcriptomic and morphological phenotypes

**DOI:** 10.1002/alz.087986

**Published:** 2025-01-03

**Authors:** Katherine E Prater, Shannon Rose, Corbin S.C. Johnson, Sam Strohbehn, Alexandra J Cochoit, Sainath Mamde, Wei Sun, C. Dirk Keene, Ali Shojaie, Gwenn A Garden, Elizabeth E Blue, Jessica E. Young, Suman Jayadev

**Affiliations:** ^1^ University of Washington, Seattle, WA USA; ^2^ University of California, San Diego, San Diego, CA USA; ^3^ Fred Hutchinson Cancer Research Center, Seattle, WA USA; ^4^ University of North Carolina, Chapel Hill, NC USA; ^5^ Institute for Stem Cell and Regenerative Medicine, Seattle, WA USA

## Abstract

**Background:**

Late onset Alzheimer disease is a complex syndrome, genetically, clinically and pathogenetically heterogeneous. Genome Wide association studies have identified risk alleles for AD harboring genes in the endolysosomal network (ELN). We hypothesize that aggregate burden of these endolysosomal risk alleles impacts cell type specific ELN function, thus contributing to LOAD pathogenesis.

**Method:**

To test this hypothesis, we first developed an endolysosomal polygenic risk score (ePRS) using a curated list of established GWAS AD risk loci. We identified a cohort in the top quartile (high) and bottom quartile (low) of ePRS scores within our LOAD biobank with rich clinical and pathological phenotype data, enabling brain tissue omics and cell based functional studies from the same donor. To explore cell type specific implications of ePRS burden, we studied frozen cortical brain tissue and primary cells derived from the same set of LOAD cases.

**Result:**

We identified cell type‐specific gene expression and regulatory network shifts associated with ePRS burden. Immunohistochemistry revealed that high ePRS was associated with enlarged neuronal endosomes and microglial lysosomes. We explored whether ePRS itself predicted ELN changes in cells that are not exposed to pathologic protein deposition. Leptomeningeal cells isolated from the ePRS cohort also manifested changes to ELN morphology and bulk RNA sequencing revealed ePRS‐dependent gene expression changes, suggesting that ePRS directly influences ELN related transcriptomic phenotype.

**Conclusion:**

Taken together, our data suggests that AD risk loci implicating genes within the endolysosomal system do in fact impact the cellular ELN, giving more confidence to the pathogenic relevance of “endolysosomal” risk loci. Dysfunction of endolysosomal pathways likely contributes to AD pathogenesis in LOAD and may highlight more specific points of intervention. Pathway‐specific polygenic risk scores can enhance our understanding of the complex mechanisms contributing to AD and pave the way for more specific targeted therapies in the clinic.

